# Routine Inflammatory and Hematological Markers and Acute Radiation Toxicity in Patients with Prostate Cancer

**DOI:** 10.3390/curroncol33080479

**Published:** 2026-08-14

**Authors:** Jolanta Łuniewska-Bury, Piotr Leśniak, Adam Majchrzak, Kamil Obel, Tomasz W. Kaminski, Tomasz Ząbkowski, Tomasz Syryło

**Affiliations:** 1 Faculty of Medicine, Collegium Medicum, The Mazovian University in Płock, 2 Pl. Dąbrowskiego Street, 09-402 Płock, Poland; 2 Provincial Integrated Hospital in Płock, 19 Medyczna Street, 09-400 Płock, Poland; 3 Department of General, Functional and Oncological Urology, Military Institute of Medicine-National Research Institute, 128 Szaserów Street, 04-141 Warsaw, Poland; 4 Warsaw Bar Association, 15/16 Żytnia Street, 01-014 Warsaw, Poland; 5 Hemostasis and Thrombosis Program, Versiti Blood Research Institute, Milwaukee, WI 53226, USA

**Keywords:** prostate cancer, hypofractionated radiotherapy, acute pelvic toxicity, inflammatory biomarkers

## Abstract

Background: Modern radiotherapy techniques have improved the safety of prostate cancer treatment, but acute gastrointestinal and genitourinary toxicity remains a common clinical problem. Early identification of patients who are likely to develop treatment-related adverse effects may help optimize supportive care. This study evaluated the relationships between clinical characteristics, radiotherapy planning parameters, systemic inflammatory biomarkers, and acute pelvic toxicity during hypofractionated radiotherapy. Materials and Methods: We retrospectively analyzed 142 patients with histologically confirmed prostate cancer who underwent external beam radiotherapy at the Provincial Integrated Hospital in Płock, Poland, between October 2024 and March 2026. Clinical, laboratory, and radiotherapy planning data were obtained from institutional electronic records. Laboratory analyses included CRP, creatinine, total leukocyte, neutrophil, lymphocyte, erythrocyte and platelet counts, hemoglobin, and hematocrit. Rectal and bladder toxicity was prospectively graded according to the RTOG/EORTC criteria at 2 and 4 weeks after treatment initiation. Correlation and multivariable regression analyses were performed to identify factors associated with acute treatment-related toxicity. Results: Radiotherapy significantly reduced leukocyte and lymphocyte counts (both *p* < 0.001) and produced smaller reductions in erythrocyte count, hemoglobin, hematocrit, and absolute neutrophil count (all *p* < 0.05). Patients who reported rectal or urinary symptoms early during treatment generally continued to experience toxicity later in the treatment course. Rectal and bladder toxicities were positively correlated, indicating that patients with toxicity affecting one pelvic organ frequently developed symptoms in the other. Routine inflammatory biomarkers and most dosimetric parameters showed limited independent value for predicting clinically relevant acute toxicity. Conclusions: Early gastrointestinal and genitourinary toxicity appears to be a useful clinical indicator of persistent treatment-related symptoms during hypofractionated prostate radiotherapy. Early rectal and bladder toxicity was associated with toxicity later during treatment, whereas routine laboratory markers and most dosimetric parameters showed limited independent associations with acute pelvic toxicity.

## Simple Summary

Radiotherapy is an effective treatment for prostate cancer, but many patients experience bowel or urinary side effects that can affect their comfort and quality of life. Identifying patients who are more likely to develop these problems early during treatment could help improve supportive care. In this study, we investigated whether routine blood tests, radiotherapy planning parameters, and early treatment-related symptoms could help identify patients at greater risk of developing acute side effects. We found that patients who developed bowel or urinary symptoms early were more likely to experience the same problems later during radiotherapy. In contrast, routine blood tests and most treatment planning parameters provided little additional predictive value. These findings suggest that paying close attention to symptoms during the first weeks of radiotherapy may provide a simple and practical way to identify patients who would benefit from closer monitoring and earlier supportive care.

## 1. Introduction

Cancer remains a major global health burden, with an estimated 20.6 million new cases and 9.8 million cancer-related deaths worldwide in 2024. Prostate cancer is the second most commonly diagnosed malignancy in men, accounting for approximately 1.5 million new cases and 420,000 deaths globally [1,2]. Its development is influenced by age, inherited susceptibility, family history, ancestry, and environmental and lifestyle factors. External beam radiotherapy is a standard treatment for patients with localized and locally advanced prostate cancer. The widespread introduction of intensity-modulated radiotherapy (IMRT), volumetric-modulated arc therapy (VMAT), and image-guided radiotherapy (IGRT) has substantially improved dose delivery, allowing for better protection of surrounding healthy tissues while maintaining excellent oncological outcomes [3,4,5,6]. At the same time, the increasing use of moderate and ultra-hypofractionated schedules has reduced the overall treatment time and improved convenience for patients without compromising treatment efficacy [6,7,8,9]. Despite substantial progress in radiotherapy, gastrointestinal and genitourinary toxicity remains a frequent complication of pelvic irradiation and continues to affect many patients treated for prostate cancer [3,6,10]. Acute radiation-induced injury to the rectum and urinary bladder can impair quality of life during treatment and may also contribute to the development of persistent or late adverse effects [10,11]. Although modern radiotherapy techniques have improved the protection of surrounding normal tissues, symptoms such as urinary urgency, proctitis, diarrhea, rectal discomfort, and cystitis are still commonly observed during treatment. Emerging evidence also suggests that acute toxicity may represent an early manifestation of individual radiosensitivity rather than a temporary treatment-related event, making it a potential indicator of subsequent radiation-induced complications [6,10,12,13].

Over the past decade, numerous studies have sought to identify factors associated with gastrointestinal and genitourinary toxicity following prostate radiotherapy. The available evidence suggests that the risk of treatment-related toxicity is influenced by both treatment- and patient-related factors. These include dose–volume histogram parameters, fractionation schedules, irradiated organ volumes, radiation dose delivered to the rectum and bladder, as well as individual clinical characteristics. In particular, the radiation dose distribution within the rectal wall and bladder has been repeatedly associated with the development of clinically relevant toxicity after both prostate bed irradiation and stereotactic body radiotherapy. Despite these advances, predicting which patients will develop toxicity remains challenging. Although many predictive models have been proposed, their performance has varied considerably, and relatively few have been successfully validated in independent patient cohorts or across different radiotherapy protocols [3,10,14]. In addition to improvements in radiotherapy planning and dosimetric optimization, growing interest has focused on the biological effects of pelvic irradiation. Ionizing radiation triggers a complex tissue response involving immune activation, endothelial damage, oxidative stress, and the release of inflammatory cytokines. Together, these processes are thought to contribute to both local radiation-induced toxicity and systemic inflammatory responses [15,16,17]. These biological changes have prompted interest in circulating biomarkers, including C-reactive protein (CRP), interleukins, transforming growth factor-β, and other inflammatory mediators, as potential markers of individual radiosensitivity and treatment-related toxicity. Recent studies suggest that changes in these biomarkers may be associated with radiation dose, irradiated tissue volume, and the development of gastrointestinal and genitourinary toxicity during treatment. Although the available evidence is promising, their role in routine clinical practice has yet to be established [3,5,12,13,15].

Most previous studies evaluated the dosimetric factors and inflammatory biomarkers separately. Only a limited number have combined clinical characteristics, laboratory findings, and radiotherapy planning parameters within a single predictive model [3,15]. In addition, relatively little is known about whether acute pelvic toxicity persists throughout hypofractionated radiotherapy or whether systemic inflammatory changes occur alongside the simultaneous development of rectal and bladder toxicity.

Previous studies have demonstrated that radiotherapy may induce systemic inflammatory changes. For example, Bower et al. evaluated circulating CRP and selected cytokine-related markers in relation to fatigue during radiotherapy in patients with breast or prostate cancer [18]. However, that study focused on fatigue rather than organ-specific pelvic toxicity and did not integrate longitudinal gastrointestinal and genitourinary toxicity assessments with radiotherapy planning parameters. Moreover, evidence regarding the clinical value of routinely available inflammatory and hematological markers in patients receiving contemporary hypofractionated prostate radiotherapy remains limited.

Therefore, the present study aimed to determine whether routinely available inflammatory and hematological biomarkers provide clinically useful information beyond early toxicity assessment and radiotherapy planning parameters. We examined the relationships between clinical characteristics, dosimetric variables, and rectal and bladder toxicity assessed at 2 and 4 weeks during prostate radiotherapy. We also evaluated whether early toxicity persisted later during treatment and whether rectal and bladder adverse effects occurred concurrently.

## 2. Materials and Methods

This retrospective study included patients with histologically confirmed prostate cancer who underwent external beam radiotherapy at the Provincial Integrated Hospital in Płock, Poland, between October 2024 and March 2026. The study was conducted at a single institution to ensure consistency in radiotherapy planning, laboratory procedures, and toxicity assessment. All patients were treated using VMAT according to institutional protocols, and acute toxicity was assessed contemporaneously during weekly clinical visits.

Clinical, laboratory, and radiotherapy planning data were obtained from institutional electronic medical records and treatment planning systems. The study investigated the associations between radiotherapy planning parameters, systemic inflammatory biomarkers, and the occurrence of acute pelvic toxicity during radiotherapy. Radiotherapy was delivered using hypofractionated treatment protocols, with a mean total dose of 59.7 ± 0.875 Gy and a mean fraction dose of 2.82 ± 0.056 Gy administered over a mean of 22.0 ± 0.464 fractions.

Radiotherapy treatment plans were generated using contemporary conformal treatment techniques. Planning target volumes (PTVs) were defined according to the institutional radiotherapy protocols. PTV1 represented the primary treatment volume, whereas PTV2 represented an additional boost target volume when clinically indicated.

Dosimetric variables included the total radiation dose, dose per fraction, number of fractions, PTV1 dose and volume, PTV2 dose and volume, and maximum dose to the penile bulb.

Acute rectal and bladder toxicity was graded prospectively during weekly clinical visits according to the RTOG/EORTC acute radiation morbidity scoring criteria [19,20]. For the present analysis, toxicity grades recorded at 2 and 4 weeks after treatment initiation were used. Rectal and bladder toxicity was assessed separately on a scale from grade 0, indicating no symptoms, to grade 4, indicating life-threatening toxicity. The complete organ-specific grading criteria are provided in Supplementary Table S1.

Peripheral blood samples were obtained before the initiation of radiotherapy and after the completion of treatment. Data from hematological analyses included the white blood cell count, red blood cell count, hemoglobin levels, hematocrit levels, platelet count, differential leukocyte counts, and absolute neutrophil and lymphocyte counts. Serum CRP and creatinine concentrations were also evaluated as systemic inflammatory and renal biomarkers, respectively.

Hematological and biochemical parameters were measured using standard laboratory methods routinely applied in the diagnostic laboratory of our institution. Acute gastrointestinal and genitourinary toxicities were assessed during radiotherapy follow-up visits. Rectal and bladder toxicities were recorded separately at 2 and 4 weeks after treatment initiation. For correlation and regression analyses, toxicity scores were treated as continuous variables.

Continuous variables are presented as mean ± standard error of the mean, with the median and interquartile range (25th–75th percentile) reported where appropriate. Laboratory values obtained before and after radiotherapy were compared using paired statistical tests selected according to the data distribution. Spearman rank correlation analysis was used to assess associations among clinical characteristics, dosimetric parameters, laboratory biomarkers, and treatment-related toxicity scores.

Correlation matrices were generated for the entire study cohort and separately for the PTV2-positive and PTV2-negative subgroups. Multiple linear regression analyses were performed to identify factors independently associated with rectal toxicity at 2 and 4 weeks and bladder toxicity at 2 and 4 weeks. The models included patient age, total radiation dose, dose per fraction, number of fractions, PTV1 volume, PTV2 volume, maximum dose to the penile bulb, rectal and bladder toxicity scores, and baseline CRP and creatinine concentrations.

For each variable, regression coefficients (β), standard errors, 95% confidence intervals, t statistics, and *p* values were calculated. All statistical analyses were performed using GraphPad Prism version 11.0 (GraphPad Software, La Jolla, CA, USA). A two-sided *p* value < 0.05 was considered statistically significant.

Additional exploratory analyses were conducted according to the presence or absence of an additional PTV2 treatment volume. Patients were stratified into PTV2-positive and PTV2-negative subgroups, and the analyses included inflammatory biomarkers, renal function parameters, and correlations between dosimetric and laboratory variables within each subgroup.

## 3. Results

A total of 142 patients were included in the analysis. Their clinical and radiotherapy characteristics are summarized in Table 1. Changes in hematological and biochemical parameters during treatment are presented in Table 2. Additional hematological indices are presented in Supplementary Table S2. The distributions of RTOG/EORTC rectal and bladder toxicity grades at 2 and 4 weeks are shown in Table 3. The mean age of the cohort was 73.0 years, and the median total radiation dose was 60.0 Gy, delivered in 20 fractions. During treatment, both rectal and bladder toxicity scores increased over time, with higher symptom severity observed after 4 weeks than after 2 weeks. The baseline clinical and dosimetric characteristics are summarized in Table 1.

**Figure 1:**
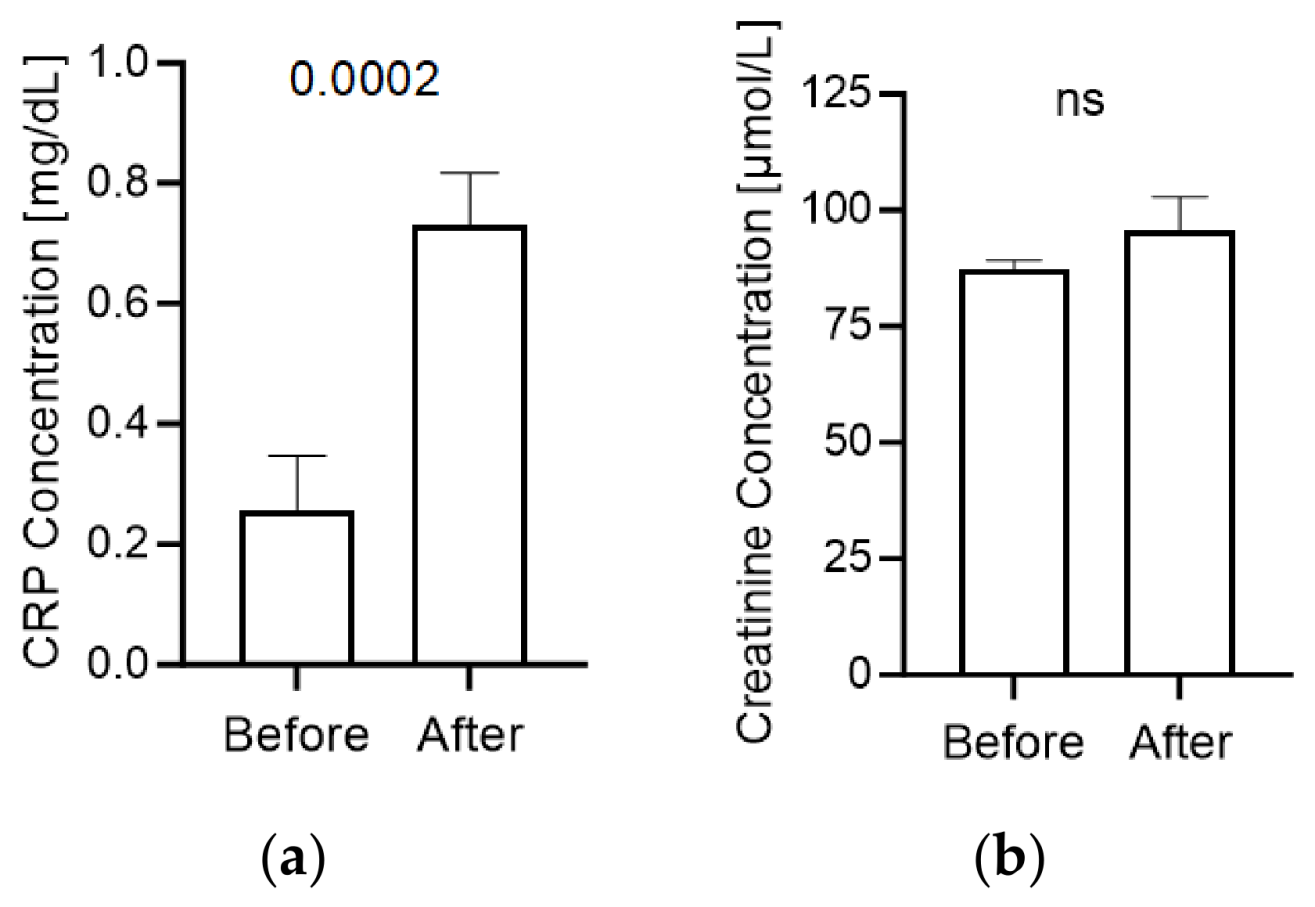
Changes in systemic inflammatory and renal biomarkers after radiotherapy. (**a**) Serum C-reactive protein (CRP) concentrations before and after treatment. (**b**) Serum creatinine concentrations before and after treatment. Data are presented as mean ± standard error of the mean. Statistical significance was assessed using paired comparisons. Abbreviation: ns, not significant.

The changes in the systemic inflammatory and renal biomarkers after radiotherapy are illustrated in Figure 1. CRP levels increased significantly after treatment (*p* = 0.0002). No significant difference in creatinine levels was observed between the pre- and post-treatment time points.

Among the 132 patients with complete toxicity data, grade ≥ 2 rectal toxicity increased from 4.5% at week 2 to 22.7% at week 4. Grade ≥ 2 bladder toxicity increased from 5.3% to 31.8% over the same period. Grade 3 toxicity was not observed at week 2 but occurred at week 4 in 6.8% of patients for rectal toxicity and 6.1% for bladder toxicity. No grade 4 rectal or bladder toxicity was recorded.

Paired pre- and post-treatment measurements demonstrated a selective systemic hematological response to radiotherapy. The white blood cell count decreased by approximately 15%, from 6.96 ± 0.214 to 5.90 ± 0.170 × 10^9^/L (*p* < 0.001). The largest relative change was observed in the absolute lymphocyte count, which decreased by approximately 33%, from 1.72 ± 0.061 to 1.15 ± 0.049 × 10^9^/L (*p* < 0.001). The absolute neutrophil count decreased by approximately 13%, from 4.50 ± 0.190 to 3.91 ± 0.141 × 10^9^/L (*p* < 0.05).

Smaller but statistically significant reductions were observed in the erythrocyte count, hemoglobin concentration, and hematocrit value (all *p* < 0.05). In contrast, platelet count, mean platelet volume, and absolute monocyte, eosinophil, basophil, and large unstained cell counts did not change significantly. CRP concentrations increased significantly after radiotherapy (*p* = 0.0002), whereas serum creatinine remained stable. Overall, the systemic response was dominated by lymphocyte depletion and an increase in the acute-phase inflammatory marker CRP, without evidence of uniform suppression across all hematological cell lineages.

**Figure 2:**
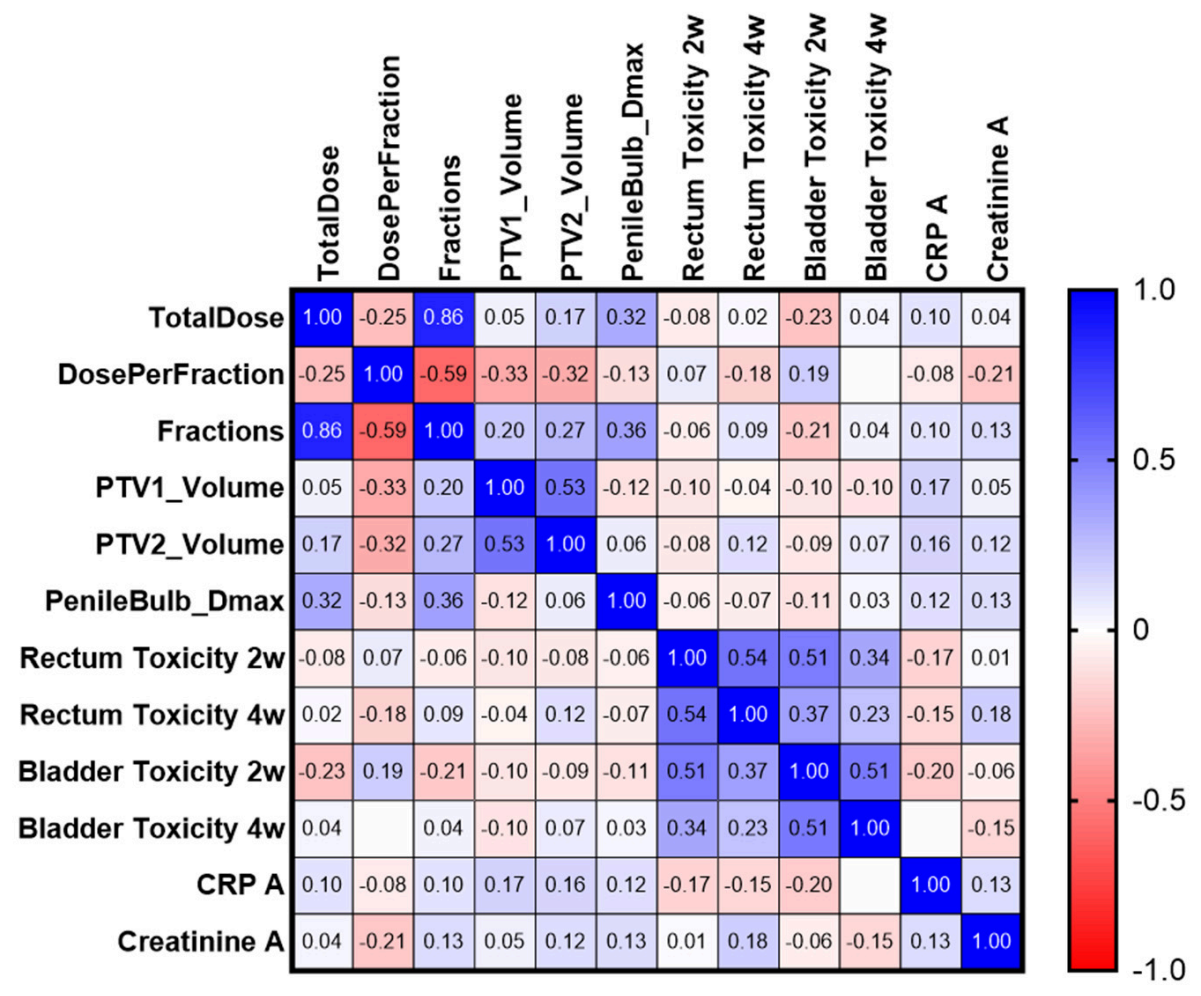
Spearman rank correlation matrix demonstrating associations between clinical characteristics, radiotherapy planning parameters, inflammatory biomarkers, and treatment-related toxicity scores in the entire study cohort. Correlation coefficients (r) are displayed within each cell. Blue is used to indicate positive correlations, whereas red is used to indicate negative correlations. A greater color intensity reflects a stronger correlation. Abbreviation: CRP, C-reactive protein; PTV, planning target volume.

The correlation analysis of the clinical, dosimetric, and laboratory parameters demonstrated several expected relationships between the radiotherapy planning parameters and treatment-related toxicities (Figure 2). Among the radiotherapy variables, a strong positive correlation was observed between the total radiation dose and number of fractions (r = 0.86), reflecting the intrinsic relationship between cumulative dose delivery and fractionation schedule. The dose per fraction was negatively correlated with the number of fractions (r = −0.59), consistent with the use of hypofractionated treatment regimens. Moderate positive correlations were also identified between PTV1 and PTV2 volumes (r = 0.53), suggesting partial overlap between treatment target volumes. Treatment-related toxicities showed moderate intercorrelations. Rectal toxicity assessed at 2 weeks after treatment initiation was positively correlated with rectal toxicity at 4 weeks after initiation (r = 0.54). Similarly, bladder toxicity at 2 weeks correlated with bladder toxicity at 4 weeks (r = 0.51). Cross-organ associations were also observed, including correlations between early rectal and bladder toxicities (r = 0.51), indicating that patients susceptible to acute radiation-induced toxicity in one pelvic organ may also exhibit increased sensitivity in adjacent tissues. The maximum dose to the penile bulb demonstrated weak to moderate positive associations with the total dose (r = 0.32) and number of fractions (r = 0.36), suggesting increased incidental exposure with more intensive treatment regimens. Few associations were observed between the inflammatory and renal biomarkers and radiotherapy parameters or toxicity scores. Only weak correlations were observed between CRP concentrations after therapy and treatment-related toxicities and dosimetric variables (all |r| < 0.20), indicating that in this cohort, the systemic inflammatory response was not strongly linked to acute pelvic toxicity. Similarly, only weak correlations were identified between the posttreatment creatinine levels and radiotherapy characteristics and toxicity outcomes, suggesting that the impact of treatment on renal function was minimal. Overall, the correlation matrix indicated that radiotherapy-associated toxicities were clustered together, whereas systemic laboratory biomarkers remained largely independent of dosimetric and clinical parameters.

Detailed results of the four multivariable regression models are presented in Supplementary Tables S3–S6. Rectal toxicity at week 2 was associated with rectal toxicity at week 4 and concurrent bladder toxicity at week 2 (Supplementary Table S3). Rectal toxicity at week 4 was strongly associated with rectal toxicity at week 2 and selected fractionation parameters (Supplementary Table S4). Bladder toxicity at week 2 was associated with concurrent rectal toxicity and bladder toxicity at week 4 (Supplementary Table S5). Bladder toxicity at week 4 was primarily associated with bladder toxicity observed at week 2 (Supplementary Table S6).

Most dosimetric variables showed limited independent predictive value for acute urinary toxicity. Similarly, inflammatory and renal biomarkers were not independently associated with clinically significant adverse effects of the rectum or bladder. Subgroup analyses based on PTV2 status demonstrated similar between-group inflammatory response patterns, with persistently weak correlations being observed between laboratory biomarkers and toxicity outcomes (Supplementary Figures S1–S3).

## 4. Discussion

Acute gastrointestinal and genitourinary toxicity during prostate radiotherapy may provide important information beyond the immediate effects of treatment. Patients who develop symptoms early in the course of radiotherapy appear to be at higher risk of persistent toxicity later during treatment or follow-up. This is especially relevant in the era of hypofractionated radiotherapy, where limiting toxicity is important not only for patient comfort but also for maintaining treatment adherence and overall treatment tolerability [6,12,13].

Patients in our cohort who developed rectal or urinary symptoms early during radiotherapy were more likely to continue experiencing toxicity later in treatment. Early toxicity may therefore serve as a simple clinical indicator of reduced treatment tolerance. Radiotherapy also led to measurable inflammatory and hematological changes, including higher CRP levels and lower lymphocyte counts. Despite these changes, only limited associations were observed between laboratory markers and clinically relevant pelvic toxicity.

Our study differs from earlier investigations linking inflammatory biomarkers primarily with general symptoms such as fatigue. We assessed organ-specific rectal and bladder toxicity and found that the increase in CRP during treatment did not translate into a strong association with acute pelvic toxicity. These findings suggest that systemic inflammation and local treatment-related symptoms may represent related but clinically distinct responses to radiotherapy.

Radiotherapy produced a selective rather than uniform systemic hematological response. The most pronounced change was lymphocyte depletion. This finding is consistent with the high radiosensitivity of circulating lymphocytes and may reflect the exposure of circulating blood cells and hematopoietic tissue during pelvic irradiation [15,16,17]. The reductions in total leukocyte and neutrophil counts were smaller, whereas platelet count and mean platelet volume remained stable. This pattern does not indicate generalized suppression of all hematopoietic lineages.

Although the reductions in erythrocyte count, hemoglobin, and hematocrit reached statistical significance, their magnitude was small. These changes may have resulted from the cumulative effects of radiotherapy, patients’ baseline clinical condition, hydration status, or other individual factors. Because anemia-related symptoms, blood transfusions, and treatment interruptions were not systematically recorded, the clinical significance of these findings remains uncertain.

The pattern of gastrointestinal and genitourinary toxicity observed in our study was similar to that reported in previous studies of hypofractionated prostate radiotherapy. Despite substantial improvements in treatment planning and dose delivery over recent years, these adverse effects remain relatively common [5,6,10]. Early toxicity assessment may have practical clinical value because it is simple, routinely available during treatment visits, and may help identify patients who could benefit from closer monitoring or earlier supportive care [12,13].

We also observed that rectal and bladder toxicity frequently occurred together during treatment. This may suggest that adverse effects in the pelvic region are not entirely isolated organ-specific events, but rather reflect shared treatment-related susceptibility patterns influenced by anatomical proximity and overlapping radiation exposure. Although age is an important risk factor for the development of prostate cancer, its role in acute radiotherapy toxicity is less clear. In the present cohort, age was included as a covariate in all multivariable models. Patient age was not independently associated with acute rectal or bladder toxicity. These findings should be interpreted cautiously because the study was not designed to evaluate disease progression, survival, or age-related differences in treatment efficacy.

Inflammatory and hematological changes were clearly detectable during radiotherapy; however, routinely available biomarkers demonstrated limited independent usefulness for predicting clinically significant toxicity. These findings support the growing view that radiation-related adverse effects are shaped by multiple interacting factors, not by isolated laboratory or dosimetric parameters alone [15,21]. Most dosimetric variables demonstrated relatively limited predictive value for acute urinary toxicity, highlighting the complexity of toxicity assessment in routine radiotherapy practice. From a practical perspective, our findings support the importance of the early monitoring of toxicity during prostate radiotherapy. Patients who develop rectal or urinary symptoms early during treatment may benefit from more individualized supportive management, including intensified follow-up, earlier symptom control, dietary recommendations, or optimization of bladder and rectal preparation protocols [6,12,13]. Earlier intervention may help improve treatment tolerability and reduce the impact of radiotherapy on the quality of life of patients.

An emerging direction in biomarker discovery is the use of biomedical foundation models. These models are pretrained on large transcriptomic or multi-omic datasets and can subsequently be adapted to tasks such as cell-state classification, data integration, gene-network inference, and the prediction of biological responses. Models such as Geneformer, scGPT, and scFoundation illustrate the potential of this approach for extracting transferable molecular representations from large-scale single-cell datasets [22,23]. In radiation toxicity research, future foundation models could integrate longitudinal inflammatory biomarkers, genomic, transcriptomic, proteomic, radiomic, dosimetric, and clinical data. Such models may identify biological patterns that are not captured by isolated markers such as CRP or peripheral blood cell counts. Nevertheless, their clinical application will require external validation, biological interpretability, and direct comparison with simpler statistical models, as current foundation models do not consistently outperform conventional approaches in all settings [24].

### Limitations

This study has several limitations. Its retrospective and single-center design limits causal interpretation and generalizability. Toxicity was assessed only during the acute treatment period, and late outcomes were unavailable. Detailed information on baseline urinary and gastrointestinal symptoms, comorbidities, medications, and supportive interventions was incomplete. Toxicity grades were ordinal, whereas linear regression was used for exploratory analyses. In addition, several dosimetric variables were interrelated, which may have reduced model stability. Only routine laboratory markers were available, without cytokine, genomic, transcriptomic, or proteomic profiling. The present study did not include genomic, transcriptomic, proteomic, or other omics datasets and therefore could not evaluate multi-omics integration or foundation-model-based biomarker discovery.

Despite these limitations, the study provides clinically relevant insight into the temporal behavior of acute pelvic toxicity during prostate radiotherapy and contributes additional evidence regarding the complex relationship between dosimetric variables, systemic inflammation, and treatment-related adverse effects. The findings support the concept that acute pelvic toxicity is not exclusively dose-dependent, but likely reflects a multidimensional interaction between treatment characteristics and individual biological susceptibility.

## 5. Conclusions

Early rectal and bladder toxicity after 2 weeks of hypofractionated prostate radiotherapy was associated with toxicity observed after 4 weeks. Rectal and bladder adverse effects also frequently occurred together, indicating that early symptom assessment may help identify patients requiring closer monitoring during treatment [6,12,13].

Radiotherapy was associated with increased CRP levels and reductions in several hematological parameters. However, routine laboratory markers and most dosimetric variables showed limited independent associations with acute pelvic toxicity [3,5,10,15,21]. These findings should be confirmed in prospective multicenter studies with standardized baseline assessment and longer follow-up.

## Figures and Tables

**Table 1:** Baseline clinical, tumor-related, and radiotherapy characteristics of the study cohort (*N* = 142).

Characteristic	Category	Value
**Age, years**		73.4 ± 6.01
**Radiotherapy setting**	Prostate-only RT	102 (71.8%)
	Prostate and pelvic lymph-node RT	19 (13.4%)
	EBRT plus brachytherapy	11 (7.7%)
	Postoperative/salvage RT	10 (7.0%)
**Radiotherapy technique**		VMAT, 142 (100.0%)
**Total radiation dose, Gy**		60.0 (60.0–70.2)
**Dose per fraction, Gy**		3.00 (2.60–3.00)
**Number of fractions**		20.0 (20.0–27.0)
**Main fractionation schedules**	60 Gy/20 fractions	68 (47.9%)
	70.2 Gy/27 fractions	33 (23.2%)
	39 Gy/15 fractions	9 (6.3%)
	Other schedules	32 (22.5%)
**PTV1 volume, cm^3^**		171 (135–285)
**Additional PTV2**		28 (19.7%)
**PTV2 volume, cm^3^**		170 (143–221), among PTV2-positive patients
Gleason score *	4 (2 + 2)	2 (1.5%)
	6 (3 + 3)	31 (23.5%)
	7 (3 + 4)	28 (21.2%)
	7 (4 + 3)	20 (15.2%)
	8 (4 + 4)	47 (35.6%)
	9 (4 + 5)	4 (3.0%)
Clinical T stage *	T1	1 (0.8%)
	T2	89 (67.4%)
	T3	42 (31.8%)
Clinical N stage *	N0	123 (93.2%)
	N1	9 (6.8%)
Clinical M stage *	M0	129 (97.7%)
	M1	3 (2.3%)
WHO performance status before RT *	0	84 (63.6%)
	1	39 (29.6%)
	2	9 (6.8%)
Androgen deprivation therapy *	Yes	72 (54.5%)
	No	60 (45.5%)

Data are presented as *n* (%) unless otherwise indicated. Age is shown as mean ± standard deviation; other continuous variables are shown as median (interquartile range). * Data available for 132 patients. The three most frequent fractionation schedules are listed separately; all remaining schedules were grouped as “other”. Abbreviations: EBRT, external-beam radiotherapy; PTV, planning target volume; RT, radiotherapy; VMAT, volumetric-modulated arc therapy; WHO, World Health Organization.

**Table 2:** Baseline laboratory characteristics of the included patients.

Parameter	Unit	Before Radiotherapy	After Radiotherapy	Significance
White blood cells (WBC)	×10^9^/L	6.96 ± 0.214 (6.70, 5.63–7.73)	5.90 ± 0.170 (5.57, 4.75–6.82)	***
Red blood cells (RBC)	×10^12^/L	4.64 ± 0.043 (4.61, 4.36–4.96)	4.48 ± 0.045 (4.49, 4.22–4.78)	*
Hemoglobin (HGB)	g/dL	14.2 ± 0.123 (14.2, 13.3–15.2)	13.8 ± 0.134 (14.0, 13.0–14.7)	*
Hematocrit (HCT)	%	42.5 ± 0.358 (42.4, 40.1–45.0)	41.2 ± 0.391 (41.6, 39.1–43.4)	*
Platelets (PLT)	×10^9^/L	226 ± 6.96 (220, 177–268)	213 ± 5.59 (209, 168–244)	ns
Mean platelet volume (MPV)	fL	8.56 ± 0.248 (8.05, 7.50–8.88)	8.29 ± 0.114 (8.10, 7.40–8.80)	ns
Absolute neutrophil count	×10^9^/L	4.50 ± 0.190 (4.25, 3.58–5.12)	3.91 ± 0.141 (3.63, 3.04–4.64)	*
Absolute lymphocyte count	×10^9^/L	1.72 ± 0.061 (1.75, 1.28–2.20)	1.15 ± 0.049 (1.11, 0.830–1.44)	***
Absolute monocyte count	×10^9^/L	0.461 ± 0.018 (0.440, 0.370–0.510)	0.432 ± 0.014 (0.420, 0.360–0.500)	ns
Absolute eosinophil count	×10^9^/L	0.210 ± 0.020 (0.160, 0.080–0.280)	0.217 ± 0.015 (0.190, 0.120–0.300)	ns
Absolute basophil count	×10^9^/L	0.028 ± 0.002 (0.030, 0.020–0.040)	0.025 ± 0.002 (0.020, 0.010–0.030)	ns
Absolute LUC count	×10^9^/L	0.159 ± 0.008 (0.145, 0.120–0.173)	0.150 ± 0.008 (0.140, 0.100–0.180)	ns

**Note:** Data are presented as mean ± standard error of the mean, with median and interquartile range (25th–75th percentile) in parentheses. Statistical significance refers to paired comparisons between measurements obtained before and after radiotherapy. * *p* < 0.05; *** *p* < 0.001; ns, not significant. LUC, large unstained cells.

**Table 3:** Distribution of acute RTOG/EORTC rectal and bladder toxicity grades at 2 and 4 weeks after treatment initiation.

Toxicity Grade	Rectal Toxicity, Week 2 (*n* = 132)	Rectal Toxicity, Week 4 (*n* = 132)	Bladder Toxicity, Week 2 (*n* = 132)	Bladder Toxicity, Week 4 (*n* = 132)
Grade 0	76 (57.6%)	56 (42.4%)	75 (56.8%)	42 (31.8%)
Grade 1	50 (37.9%)	46 (34.8%)	50 (37.9%)	48 (36.4%)
Grade 2	6 (4.5%)	21 (15.9%)	7 (5.3%)	34 (25.8%)
Grade 3	0 (0.0%)	9 (6.8%)	0 (0.0%)	8 (6.1%)
Grade 4	0 (0.0%)	0 (0.0%)	0 (0.0%)	0 (0.0%)

Values are *n* (% of evaluable patients). Toxicity data were available for 132 out of 142 patients and were unavailable for 10 patients. Rectal and bladder toxicity was graded prospectively during weekly clinical visits using the RTOG/EORTC scale. Abbreviations: EORTC, European Organization for Research and Treatment of Cancer; RTOG, Radiation Therapy Oncology Group.

## Data Availability

The data supporting the findings of this study are available from the corresponding author upon reasonable request.

## Supplementary Materials

The following supporting information can be downloaded at: https://www.mdpi.com/article/10.3390/curroncol33080479/s1, Table S1: RTOG/EORTC criteria used for grading acute rectal and bladder toxicity; Table S2: Expanded hematological characteristics before and after radiotherapy; Tables S3: Multivariable linear regression analysis of factors associated with rectal toxicity at week 2; Tables S4: Multivariable linear regression analysis of factors associated with rectal toxicity at week 4; Table S5: Multivariable linear regression analysis of factors associated with bladder toxicity at week 2; Table S6: Multivariable linear regression analysis of factors associated with bladder toxicity at week 4; Figure S1: Comparison of inflammatory and renal biomarkers in patients with and without PTV2; Figure S2: Spearman correlation analysis in the PTV2-positive subgroup; Figure S3: Spearman correlation analysis in the PTV2-negative subgroup.

## Abbreviations

The following abbreviations are used in this manuscript:

CRP	C-reactive protein
PTV	Planning target volume
